# Hydrogel-based formulations for urothelial cancer therapy

**DOI:** 10.3389/fphar.2024.1478394

**Published:** 2024-09-25

**Authors:** Mingyang Chang, Changliang Chi, Zuozhu Zheng, Ming Zhang, Jianing Lv, Xiaoqing Wang

**Affiliations:** The First Hospital of Jilin University, Changchun, China

**Keywords:** hydrogel, urothelial cancer, bladder cancer, drug, delivery, therapy

## Abstract

Drug infusion therapy after surgery for urothelial carcinoma is an effective measure to reduce cancer recurrence rate. Hydrogels are drug carriers with good biocompatibility and high drug loading capacity, which can optimize the pharmacokinetics of drugs in the urinary system to improve the therapeutic effect. Compared with the traditional free drug *in situ* perfusion, the hydrogel drug loading system can still maintain effective drug concentration in the face of continuous urinary flushing due to its good mucosal adhesion effect. The significantly prolonged drug retention time can not only improve the therapeutic effect of drugs, but also reduce the discomfort and risk of urinary tract infections caused by frequent drug infusion, and improve patient compliance. In addition, the combination of hydrogel with nanoparticles and magnetic materials can also improve the mucosal permeability and targeting effect of the hydrogel drug loading system, so as to overcome the mucus layer of urinary epithelium and the physiological barrier of tumor and minimize the impact on normal tissue and cell functions. At present, the research of hydrogels for urothelial cancer treatment involves chemotherapy, immunotherapy, gene therapy, inhibition of metabolism and multi strategy synergistic therapy. This review summarizes the research progress of hydrogels for the treatment of urothelial carcinoma, hoping to provide a reference for the future research of safe, reliable, effective, and advanced hydrogels with little side effects.

## 1 Introduction

**Scheme 1:**
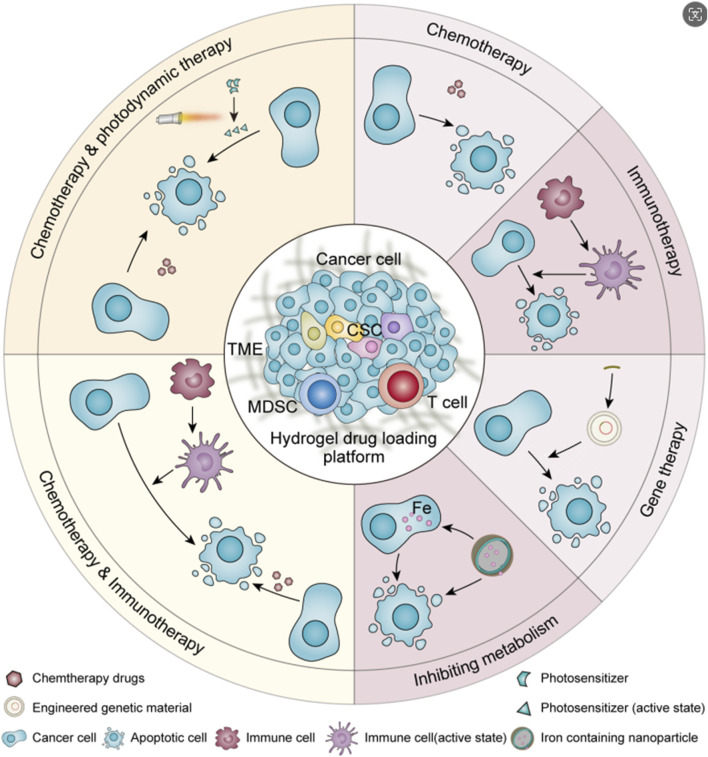
Treatment strategy of hydrogel-based formulations for urothelial carcinoma.

Urothelial carcinoma is one of the top ten malignant tumors, among which bladder cancer is the most common (Sung et al., 2021). With the progress of treatment technology, the mortality rate of urothelial carcinoma has greatly decreased, but the high recurrence rate of urothelial carcinoma after surgery has always been a problem that troubles clinicians (Rouprêt et al., 2023; Compérat et al., 2022). At present, the most common treatment for urothelial carcinoma in clinical practice is still radical surgical resection or resection of part of the lesion (Pfail et al., 2021; Leow et al., 2019; Gschwend et al., 2019; Lerner and Svatek, 2019; Peyronnet et al., 2019), which can be supplemented with postoperative chemotherapy to reduce the postoperative recurrence rate (Messing et al., 2018; Sylvester et al., 2016; Babjuk et al., 2022; Koch et al., 2021). For some patients with localized lesions and low malignancy or who are not suitable for surgery for various reasons, ureteroscopic endoscopic ablation (Villa et al., 2016), thermosensitive hydrogel chemical ablation (Matin et al., 2022), chemotherapy (Leow et al., 2021; Freifeld et al., 2020; Birtle et al., 2020), immunotherapy (Necchi et al., 2022; Bajorin et al., 2021; Balar et al., 2021; Catto et al., 2021) or radiotherapy (Iwata et al., 2019; Song et al., 2021) can be used. *In situ* drug infusion therapy has become a popular method of postoperative treatment because of its small scope of action, almost no effect on the organs of the whole body, simple operation and other advantages (Lopez-Beltran et al., 2024; Yoon et al., 2020). However, in order to prolong the action time of the drug as much as possible, the bladder needs to be filled for a period of time after *in situ* drug instillation, and the continuous flow of urine into the bladder makes the pressure on the bladder wall much higher than usual, and the discomfort of the patients is more obvious (de Lima et al., 2022). And the inevitable periodic micturition behavior makes the *in situ* infusion of drugs excreted with the urine, the drug concentration decreases sharply, and the effective drug concentration is difficult to maintain (Kolawole et al., 2017). Because of the tight arrangement of urothelial cells and the mucus barrier of urothelial cancer, the permeation ability of drugs is weak, and the therapeutic effect is greatly reduced (Dalghi et al., 2020; Zhang et al., 2023). At present, in order to solve the problem of short action time of effective drug concentration in clinic, regular drug perfusion is often used to achieve a certain therapeutic effect. The enhancement of drug permeability and the realization of drug selectivity have not been fully considered in the clinical regimen. Therefore, an accurate and efficient drug delivery strategy with good sustained release effect, strong permeation ability and targeting tumor is the research focus of *in situ* perfusion therapy for urothelial cancer. Because of its good biocompatibility and high drug encapsulation efficiency, machinable modified hydrogel is a good drug delivery carrier for *in situ* drug infusion after urothelial cancer operation. It is a promising way to solve the obstacle of *in situ* drug delivery in urothelial cancer (Wu et al., 2024; Nadler et al., 2021). This article reviewed the research progress of drug carrying strategies of hydrogel in chemotherapy, immunotherapy, gene therapy, interference metabolism therapy and multiple strategies for urothelial cancer in recent years, and summarized the solutions to the problems of continuous release, blockage, penetration and targeting of drug perfusion in the urinary system(Scheme 1). It is expected to provide a reference for the design of hydrogels with higher safety and better therapeutic effect, or have some enlightenment significance for the development and optimization of *in situ* treatment of urothelial carcinoma.

## 2 Single therapeutic strategy

### 2.1 Chemotherapy

Hydrogel encapsulated chemotherapeutic drugs is one of the most extensive therapeutic strategies for *in situ* drug perfusion of urinary system, including mitomycin, doxorubicin, epirubicin, gemcitabine, cisplatin, paclitaxel and so on. Intelligent hydrogels can achieve effective mucosal adhesion through physical or chemical cross-linking, which can not only significantly prolong the action time of the drug, but also maintain the local effective drug concentration (Qiu et al., 2020). Liu et al. delivered paclitaxel using chitosan nano-gel. The positively charged properties of chitosan make it easy to adhere to the bladder mucosa through electrostatic adsorption, and the slow release of paclitaxel has a residence time of more than 10 days (Liu et al., 2018). Radical nephrectomy is often used to treat most upper urinary tract urothelial carcinoma, which makes the patients with low-grade upper urinary tract urothelial carcinoma have to remove the diseased side of the kidney. The operation causes great damage to the body and is easy to cause renal insufficiency and many other complications. The hydrogel drug delivery system with good sustained release effect solves this problem, enables *in situ* drug infusion of upper urinary tract urothelial cancer to be realized, and gets rid of the problem that it is difficult to carry out frequent *in situ* drug infusion in upper urinary tract because of the long and narrow passageway. and greatly reduce the risk of urinary tract infection. K et al. conducted a phase 3 clinical trial in which UGN-101, a thermosensitive hydrogel containing mitomycin C, was infused into the renal pelvis *in situ* to treat low-grade urothelial carcinoma of the upper urinary tract. After 6 weeks of regular drug infusion, nearly half of the patients achieved complete remission, while other patients achieved complete remission to varying degrees (Kleinmann et al., 2020). R et al. carried out antegrade renal pelvis *in situ* drug perfusion therapy by means of nephrostomy, which reduced the occurrence of ureteral stricture and improved the comfort of patients. Reducing the effect of percutaneous nephrostomy on kidney is a problem to be solved in this approach (Rosen et al., 2022). Indeed, the use of hydrogels significantly prolonged the duration and concentration of the drug. However, this alone is not enough. How to further improve the efficacy and reduce complications is the direction of its development.

**FIGURE 1:**
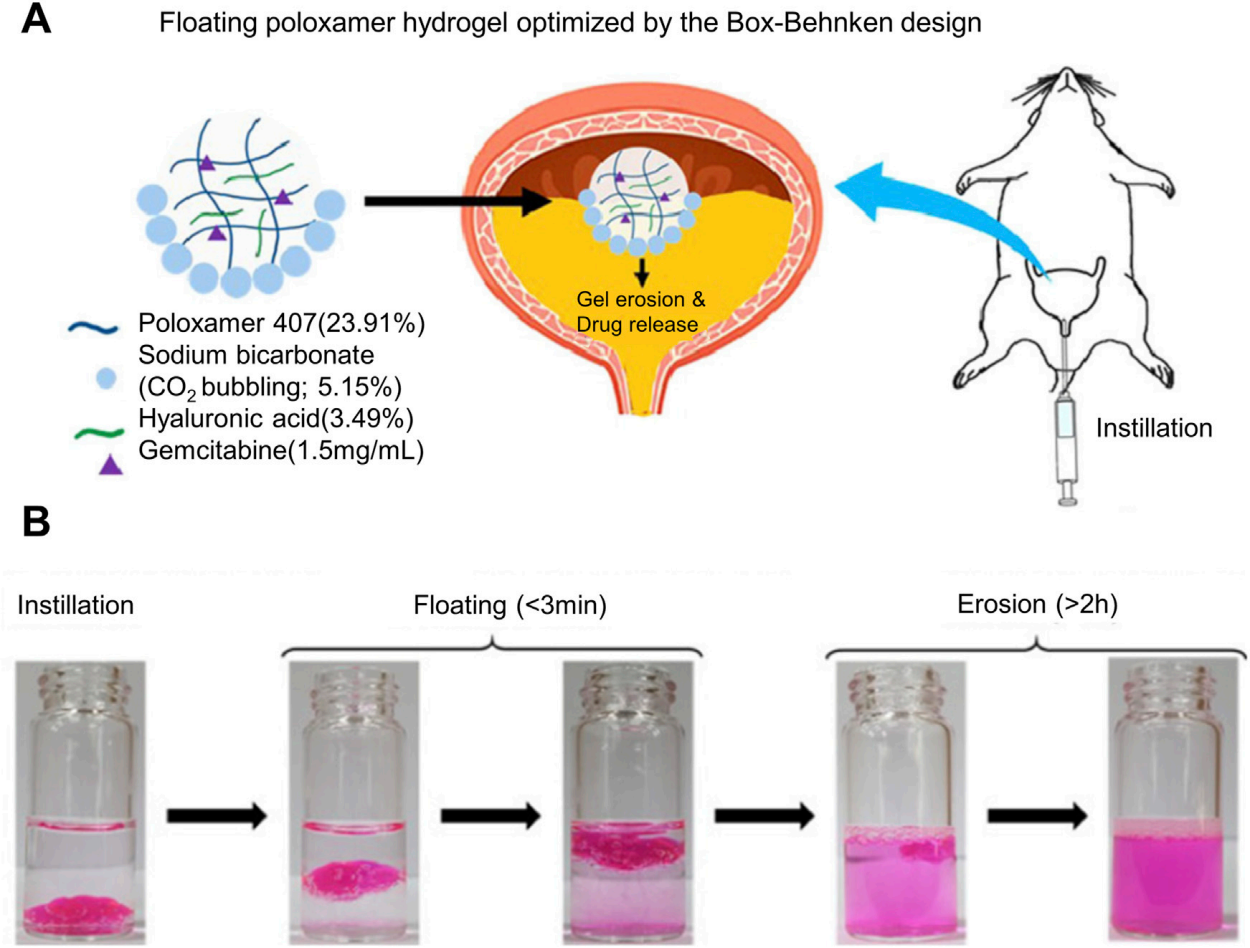
Poloxamer407-NaHCO3 floating hydrogel drug delivery system for bladder drug delivery. **(A)** Schematic diagram of NaHCO3 as a floating agent and hyaluronic acid as a gel strength regulator of Poloxamer407 thermosensitive hydrogel that can float on urine in the bladder for the delivery of Gemcitabine. **(B)** *In vitro* simulation of the floating performance of thermosensitive hydrogel, using Rhodamine B as a dye to visually display the drug release effect.

When drug-loaded hydrogel is injected into the urinary system, it may enter the relatively narrow part of the urinary tract and cause urinary tract obstruction due to changes in body position and cyclic urination, thus causing a series of problems. In order to improve the problem of gel blocking the urinary tract, researchers have proposed a drug delivery strategy that makes hydrogel float on the liquid surface, reducing its own density by generating microbubbles in the hydrogel. When the density is lower than that of urine, Realize the floating of drug-loaded hydrogel, thereby greatly reducing the probability of urinary tract obstruction. Most of the current hydrogel floating strategies are to mix sodium bicarbonate (NaHCO_3_), ammonium bicarbonate (NH_4_HCO_3_), perfluoropentane (PFP), etc., which can produce tiny bubbles under certain conditions, into the hydrogel. When conditions such as urine pH or temperature change, microbubbles will be generated inside the hydrogel. The difference in density allows the hydrogel drug-loaded system to float (Qiu et al., 2020). As shown in Figure 1A, G et al. added NaHCO_3_ as a floating agent to thermosensitive hydrogel Poloxamer407 (PLX) for intravesical drug delivery. With the decomposition of NaHCO_3_, the carbon dioxide microbubbles produced make the hydrogel produce a certain buoyancy, which can float on the urine in the bladder, and it is not easy to be excreted into the urethra with the urine in the periodic micturition process, thus reducing the risk of hydrogel blocking the urethra. As shown in Figure 1B, the *in vitro* simulation experiment shows that the floatable hydrogel drug loading platform can float within 3 minutes after bladder instillation, and the effective buoyancy can make the hydrogel drug loading system float for more than 2 h. It not only achieves efficient drug delivery but also effectively solves the problem of urinary tract obstruction, but how to further improve the floating time of hydrogel is the goal of further development (Goo et al., 2021). The problem of urinary tract obstruction commonly existed in hydrogel preparations has been solved, but whether the trace gas produced by hydrogel preparations has a certain impact on the normal function of the urinary system still needs to be explored.

**FIGURE 2:**
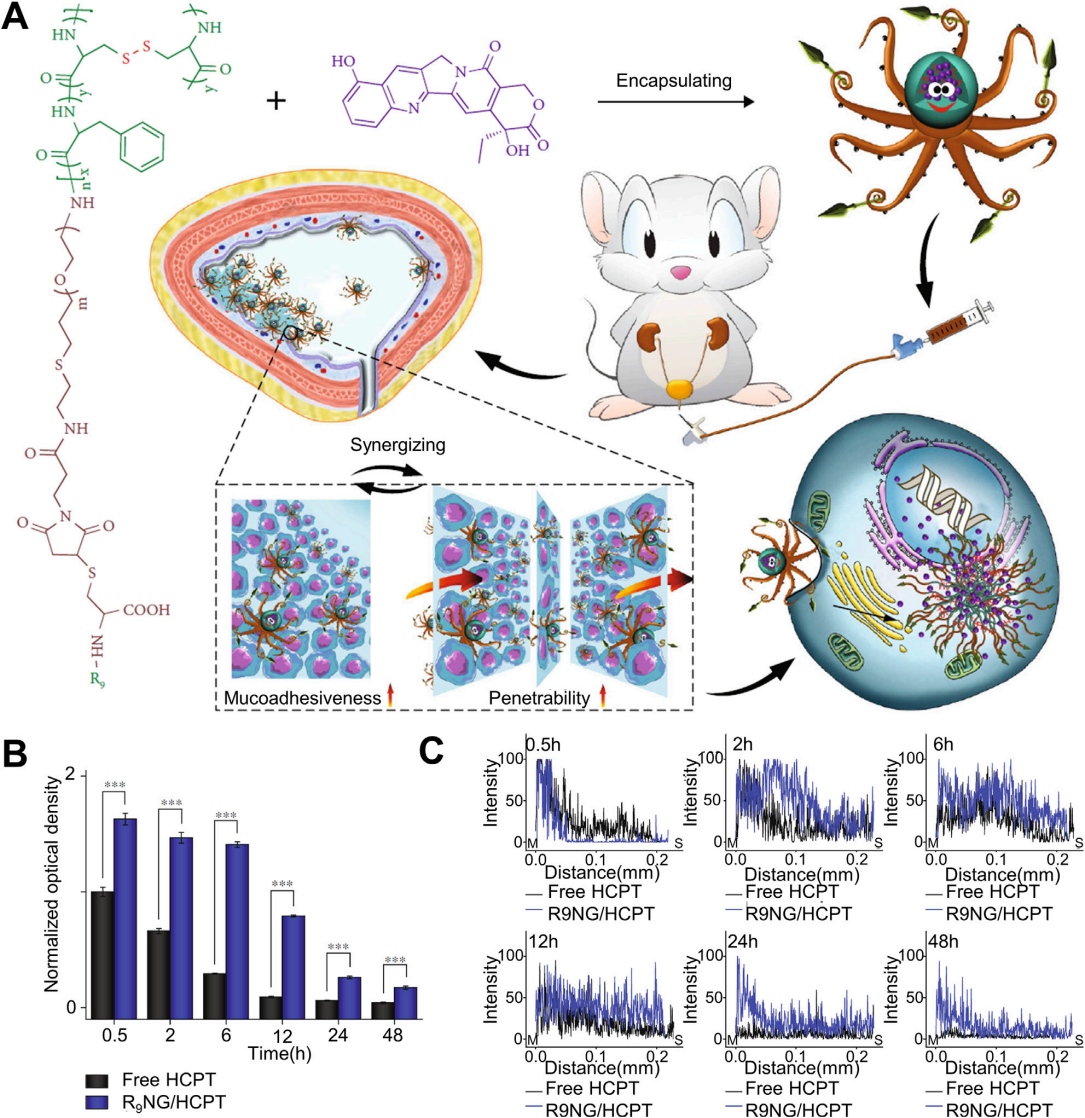
Nanogel drug delivery system for enhanced bladder mucosal penetration. **(A)** Schematic diagram of nanogel of oligoarginine-poly(ethylene glycol)–poly(L-phenylalanine-co-L-cystine) (R9-PEG–P(LP-co-LC)) prolonging drug retention time in the bladder and enhancing drug penetration into the bladder mucosa. **(B)** Quantitative results of fluorescence intensity on the mucosal surface measured by CLSM at different times when free HCPT and R9NG/HCPT adhere to the bladder mucosa. **(C)** Quantitative results of fluorescence intensity on the longitudinal section of the mucosa measured by CLSM at different times when free HCPT and R9NG/HCPT adhere to the bladder mucosa.

The penetration ability of drugs is an important issue affecting the effect of *in situ* drug infusion treatment of urothelial cancer. The mucus layer and tumor physiological barrier of urothelial cancer form a natural protective umbrella for urothelial cancer to resist the effects of local drugs, which greatly weakens the Drug penetration has a significant impact on its therapeutic effect. Overcoming the mucus barrier and tumor physiological barrier is an effective solution to improve the drug delivery efficiency of hydrogel drug-loaded systems (Zhang et al., 2023). As shown in Figure 2A. Guo et al. used oligosarginine-polyethylene glycol-poly (L-phenylalanine-co-L- cystine) nano-gel loaded with 10-hydroxycamptothecin (HCPT) for *in situ* bladder instillation. PEG improved the water dispersion of the drug and enhanced the mucosal adhesion of the nano-gel through non-specificity. The interaction between positively charged oligo-arginine and negatively charged bladder mucosa can effectively improve the permeation effect of drugs. As shown in Figure 2B, thanks to the non-specific interaction between nano-gel and bladder mucosa, *in vivo* experiments showed that free HCPT could not be detected in bladder mucosa 6 h after intravesical instillation, while the drug concentration of nano-gel loading system was 4.8 times higher than that of free drug group at 6 h, and a certain concentration of drug could be detected at 24 h, which greatly prolonged the effective time of drug action. Because the positive charge of R9 gives the nano-gel a strong permeation effect, as shown in Figure 2C, the nano-gel group can basically permeate the whole bladder layer with high drug concentration over time, while the free drug group has limited permeability and the drug concentration decreases significantly with time. The experimental results show that the nano-gel has excellent adhesion effect and permeability (Guo et al., 2020). Liposomes are widely used in the field of drug delivery because of their good mucosal adhesion. S et al. have studied a nano-gel liposome composite drug delivery system for paclitaxel delivery. The composite system simulates the lipid membrane and mucous layer that make up the urothelial barrier and enhances the adhesion and penetration (GuhaSarkar et al., 2017). C et al. added papain to mucosal adhesive hydrogel to deliver gemcitabine and used papain to destroy the mucus barrier of tumor (de Lima et al., 2023). With the help of papain, the penetration time of gemcitabine in the bladder mucosa is reduced and its penetration ability is significantly enhanced. The enhanced tissue penetration ability enhances the therapeutic effect of drugs, but the common killing effect also affects normal tissues. Further improving the targeting ability of hydrogels is a good way to solve the problem.

**FIGURE 3:**
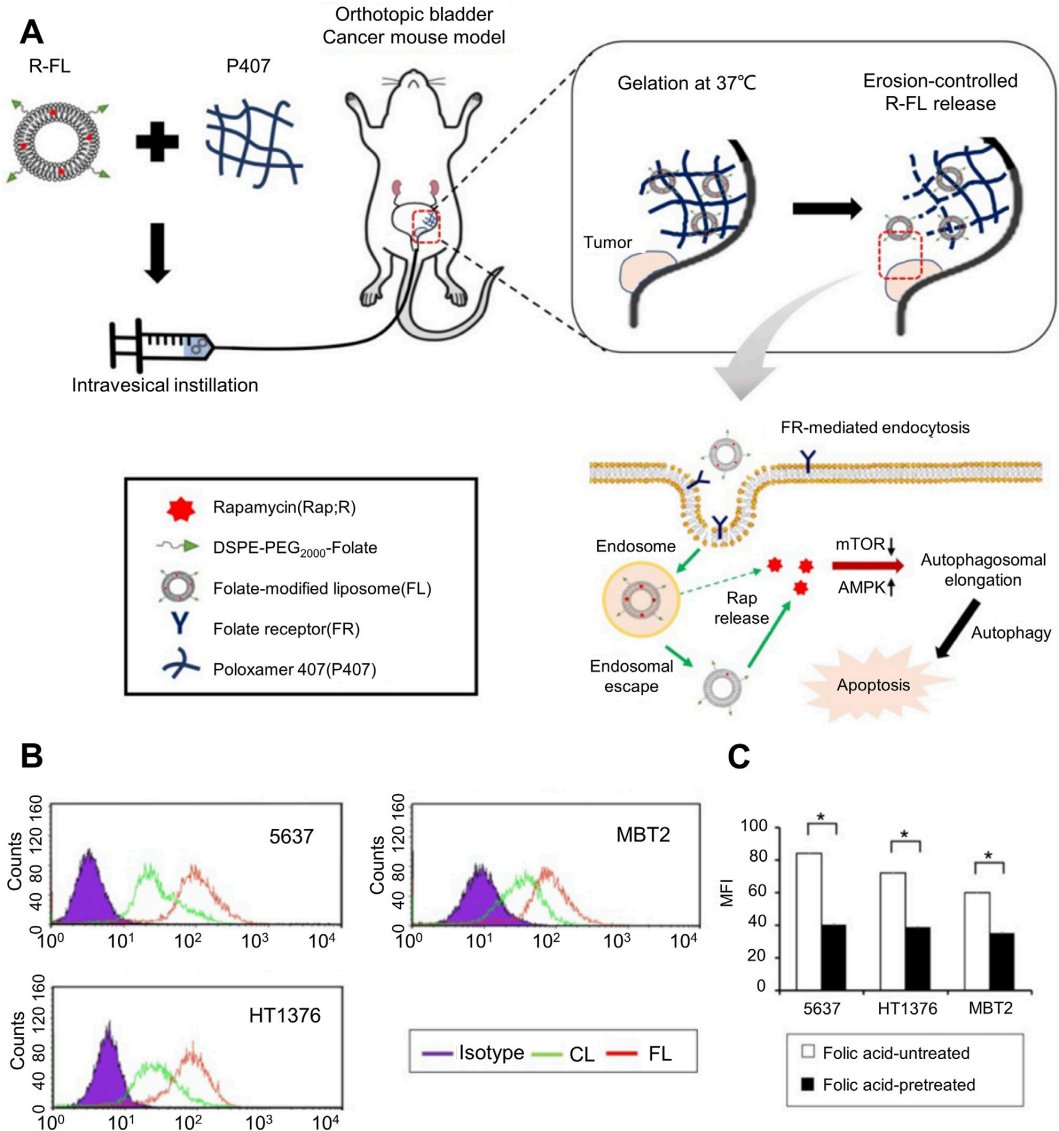
Folic acid-targeted nanogel liposome complex drug delivery system. **(A)** Schematic diagram of the action process of the targeted folate-modified nanogel liposome complex system for delivering Rap. **(B)** Quantitative analysis of the uptake of folate-modified and folate-unmodified liposomes in different bladder cancer cell lines evaluated by flow cytometry. **(C)** Competitive analysis of the internalization of folate-modified liposomes in different bladder cancer cell lines under folate-untreated and folate-pretreated conditions.

Currently, most drugs used for *in situ* infusion after urothelial cancer surgery are not selective. Therefore, normal tissue cells will also be attacked by the delivered drugs, and the normal functions of tissues and organs will also be affected to a certain extent. Enabling drugs to accurately target tumor sites or tumor cells to avoid or stay away from non-target sites as much as possible to reduce the impact on the normal metabolic activities of normal tissue cells is the ideal goal of current drug delivery. The hydrogel drug delivery system relies on its excellent loading capacity for a variety of substances and processability at the molecular level to provide attachment and binding sites for targeted substances, thereby enabling the hydrogel to have targeted release and target areas. Targeting the function of specific tissue cells, this allows high-concentration drugs to be localized to specific disease sites or cells, and the involvement of healthy areas is greatly reduced. Sun et al. incorporated magnetic Fe_3_O_4_ into chitosan hydrogel for the delivery of pirarubicin (THP). Under the action of an *in vitro* magnetic field, the hydrogel was guided to the target tumor site to achieve targeted release characteristics, which was effective The drug concentration is relatively limited to the lesion site, and the drug concentration in non-target sites is greatly reduced, preserving the normal metabolic activities of healthy tissue cells as much as possible (Sun et al., 2022). As shown in Figure 3A, H et al. used folic acid modified nano-gel liposome composite system to deliver rapamycin (Rap). After the composite drug delivery system is injected into the bladder, the liposomes gradually release from the hydrogel and are successfully taken up and internalized by bladder cancer cells under the mediation of surface folic acid, releasing the loaded Rap and achieving a tumor killing effect. The quantitative results of liposome cell uptake evaluated by flow cytometry are shown in Figure 3B. The liposome group shifted significantly to the right, especially in the folic acid modified liposome group, indicating that folic acid-mediated liposome drug loading system can significantly improve the uptake of bladder cancer cells. In order to further prove the effect of folic acid on the endocytosis of bladder cancer cells in liposome-loaded system, as shown in Figure 3C, the competitive assay of FL internalization was carried out under the condition of folic acid untreated or pretreated with folic acid. The results showed that the FR-mediated internalization was significantly inhibited by the competition between free folic acid and folic acid conjugates in the folic acid pretreatment group, and the fluorescence intensity was significantly lower than that in the untreated group. The ability of folic acid to target bladder cancer cells in drug loading system was further determined. Studies have shown that under the guidance of folic acid receptor, tumor cells significantly enhance drug endocytosis, while normal tissue cells have no corresponding targets on the surface, which can greatly reduce the impact on their normal life activities (Yoon et al., 2019). After endowing hydrogel with the ability to target tumors, the adjacent normal tissues are also protected when the target is attacked.

### 2.2 Other treatments

The application of hydrogel drug delivery systems can also be extended to immunotherapy, gene therapy, influencing tumor cell metabolism and other therapeutic strategies. Table 1 summarizes the research progress of hydrogel in the treatment of urothelial carcinoma. In order to achieve more efficient drug delivery effects, hydrogel drug delivery systems are often endowed with multiple functions at the same time. On the basis of achieving good drug encapsulation efficiency, the sustained release effect is more durable, the drug penetration ability is stronger and the target effect is more accurate.

**TABLE 1:** Summary of hydrogel-based formulations for urothelial carcinoma.

Category	Treatment strategy	Characteristics	Materials	Payload	Advantage	Limitation	Percentage of tumor reduction	References
Single therapeutic strategy	Chemotherapy	Adhesion	Chitosan	Paclitaxel	Long detention time (more than 10 days)	No selectivity	88%	Liu et al. (2018)
		Floating	Poloxamer Sodium bicarbonate	Gemcitabine	Avoid obstructing the urethra (buoyancy lasts for more than 2 h)	Gas residue	——	Goo et al. (2021)
		Infiltration	Liposome	Paclitaxel	Enhanced penetration	No selectivity	——	GuhaSarkar et al. (2017)
		Targeting	Folic acid liposome	Rapamycin	Accurate targeting	Stability uncertainty	88%	Yoon et al. (2019)
	Immunotherapy	——	Chitosan β-glycerophosphate Fe3O4 magnetic nanoparticle	BCG vaccine	Long detention time	Poor selectivity	96%	Zhang et al. (2013)
	Gene therapy	——	Chitosan-hyaluronic acid dialdehyde	SiRNA	Gene intervention	Security uncertainty	78%	Liang et al. (2021)
	Metabolic therapy	——	Hyaluronic acid	Iron oxide	Inducing ferroptosis	Security uncertainty	94%	Qi et al. (2021)
Multiple synergia therapeutic strategy	Chemotherapy +photodynamic therapy	——	PCL-PTSUO-PEG	Doxorubicin, zinc phthalocyanine	Synergia therapy	Security uncertainty	71%	Huang et al. (2018)
	Chemotherapy + Immunotherapy	——	PDLLA-PEG-PDLLA (PLEL)	Gemcitabine+ TLR9	Synergia therapy	Security uncertainty	——	Liu et al. (2024)

Immunotherapy plays a crucial role in the treatment of tumors. Therefore, the research of hydrogels is also related to immunotherapy. Zhang et al. used magnetic thermosensitive hydrogel for intravesical delivery of *Bacillus* Calmette-Guérin (BCG). Compared with traditional BCG infusion therapy, this strategy significantly extended the effective drug concentration at the tumor site and was limited to high concentrations at the tumor site as much as possible. Concentrated BCG induced a more potent Th1 immune response near the target site, significantly enhancing the anti-tumor effect (Zhang et al., 2013).

With the gradual maturity of genetic technology, human control over genes is sufficient to influence or even alter certain life activities of living organisms. Therefore, introducing genetic fragments that can inhibit oncogenes into tumor cells is a manifestation of the application of gene technology in medicine. Liang et al. covalently linked engineered small interfering RNA (SiRNA) that interferes with the Bcl2 oncogene in bladder cancer cells to chitosan-hyaluronic acid dialdehyde nanoparticles (CS-HADNPs), which can target the bladder. CD44 is overexpressed on cancer cells, and SiRNA is successfully introduced into bladder cancer cells through the interaction between ligand and receptor, and effectively inhibits the expression of its oncogene Bcl2, achieving inhibition of bladder cancer from a genetic level (Liang et al., 2021).

**FIGURE 4:**
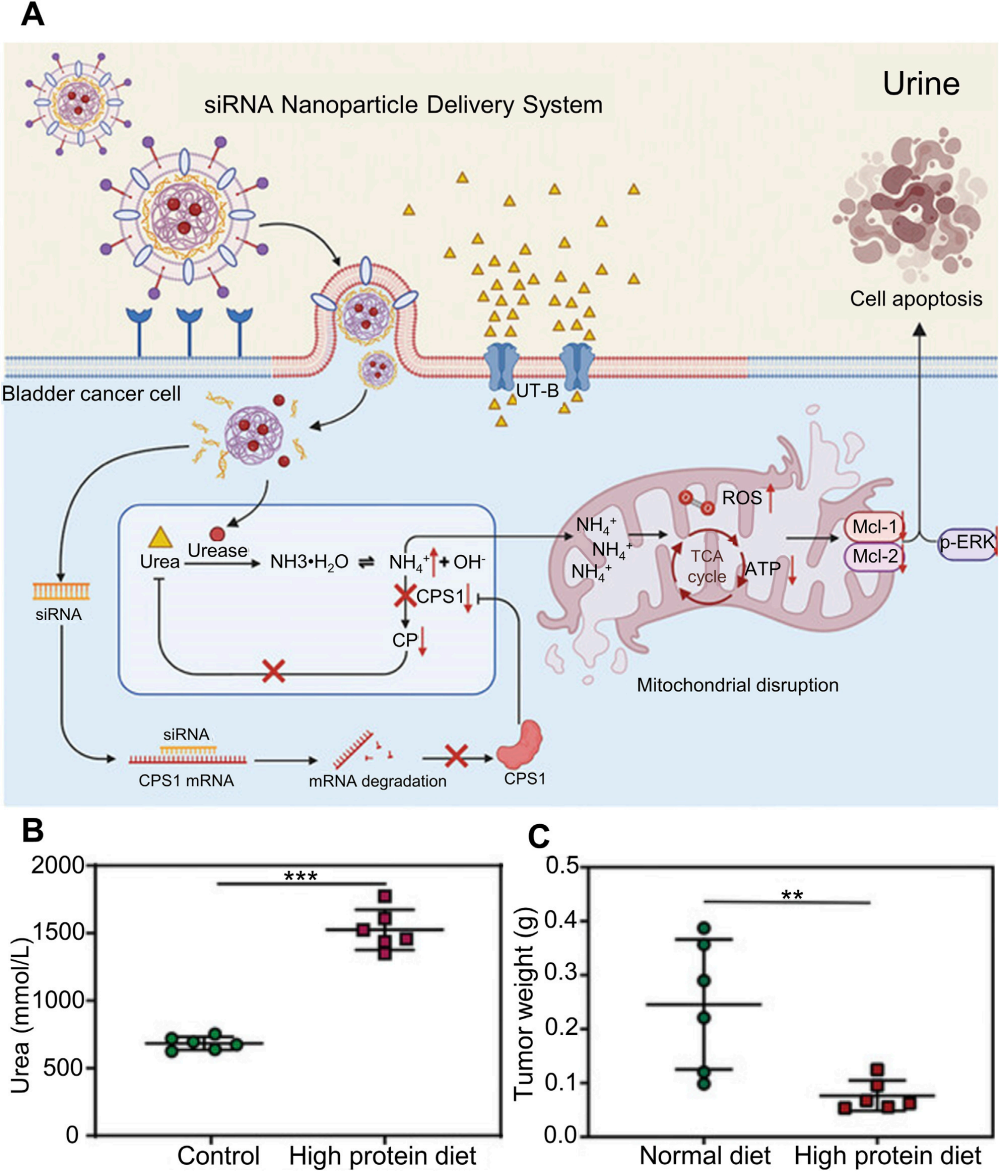
Liposomal nanotransporter hydrogel drug delivery system that can affect intracellular ammonia metabolism. **(A)** Schematic diagram of the liposomal nanotransporter hydrogel drug delivery system for targeted co-delivery of urease and siRNA that interferes with carbamoyl phosphate synthetase 1, inhibiting cellular metabolism of ammonia and increasing intracellular ammonia accumulation. **(B)** Determination of urea concentration in the urine of tumor-bearing mice under different dietary conditions. **(C)** Statistical graph of tumor weight after treatment of tumor-bearing mice under different dietary conditions.

Interfering with the high level of metabolism of tumor cells is a hot research direction at present, by reducing the efficiency of the key metabolic pathway that provides a comfortable environment for the rapid proliferation of tumor cells, so as to achieve tumor inhibition or induce apoptosis. As shown in Figure 4A, Jing et al. designed a liposome hydrogel drug delivery system for targeted co-delivery of urease and engineering siRNA that interferes with carbamyl phosphate synthase 1 (CPS1). After the hydrogel drug loading system adheres to the bladder mucosa, under the mediation of bladder cancer cell specific targeting peptide BLD-1, the liposome nano-transporter decomposes in the cytoplasm of bladder cancer cells, releasing urease and siCPS1, increasing the production of intracellular ammonia and inhibiting its normal ammonia metabolism pathway, the continuous accumulation of ammonia in bladder cancer cells, and finally high concentration of ammonia induced tumor cell apoptosis. Successfully inhibit bladder cancer cells by regulating the metabolic cascade of intracellular ammonia. In order to further verify that the drug loading system did increase the ammonia accumulation of bladder cancer cells and cause their apoptosis, as shown in Figure 4B, some tumor-bearing mice were fed a high-protein diet, and the concentration of urea in urine was significantly higher than that in the normal diet group. The tumor-bearing mice were treated with liposome nano-transporter hydrogel *in situ* under the above diet conditions, and the anti-tumor effect was shown in Figure 4C The tumor weight of tumor-bearing mice in the high-protein diet group was significantly lower than that in the normal diet group. This is because the concentration of urea in urine of tumor-bearing mice in high-protein diet group is higher, and a large amount of urea is delivered to tumor cells through urea transporter-B. The pathway of ammonia metabolism is inhibited by hydrogel drug loading system, so the ammonia accumulation in bladder cancer cells is serious and a large number of cells apoptosis (Jing et al., 2023). Ferroptosis and copper death in tumor cells have received increasing attention from researchers in recent years. Recurrent bladder cancer is somewhat resistant to *in situ* chemotherapy of the bladder, but this state is susceptible to ferroptosis. Taking advantage of this feature, Q et al. *in situ* delivered hyaluronic acid-coated iron oxide nanoparticles (IONP-HA) via a mucoadhesive hydrogel platform to increase the labile iron pool (LIP) of bladder cancer cells, benefiting from The hydrogel drug-loaded system increased the iron content in bladder cancer cells by 50 times compared with systemic administration, effectively inducing ferroptosis of bladder cancer cells (Qi et al., 2021).

The research of single therapeutic strategy hydrogel has been endowed with rich functions. But how to design a more safe and stable hydrogel with multiple characteristics still needs further research.

## 3 Multiple synergia therapeutic strategy

An inevitable problem faced by drug treatment is the decrease in sensitivity of tissue cells to drugs. The phenomenon of drug resistance is more prominent under single drug treatment. Similarly, in the treatment of tumors, there are also problems such as poor response to single-strategy tumor drug treatment. Thanks to the ability of hydrogels to co-load drugs, multi-strategy synergistic drug therapy can be better realized. Multi-strategy drug combination therapy can also complement each other to achieve synergistic treatment effects on the basis of exerting tumor suppressive functions through different pathways. The problem of reduced drug sensitivity is greatly reduced, and the tumor suppressive effect is more prominent.

Photodynamic therapy has received widespread attention as a safe, controllable, and effective treatment method. Thus, its combination with chemotherapy has a good synergistic effect on tumor killing. Z et al. used a hydrogel drug-loading platform to jointly load doxorubicin (DOX) and zinc phthalocyanine (ZnPC) in the bladder *in situ*. The synergistic treatment of chemotherapy and photodynamic therapy (PDT) showed better efficacy than single-strategy treatment. Strong tumor suppressive effect (Huang et al., 2018).

Chemotherapy and immunotherapy are the most common treatment strategies for cancer. In some cases, single strategy medication can lead to drug tolerance in tumor cells. So, considering the simultaneous application of chemotherapy and immunotherapy is a strong and effective synergistic approach. Liu et al. used thermo-sensitive hydrogel to deliver gemcitabine (GEM) *in situ* into the bladder to directly kill tumors, and also used the sustained-release ability of thermo-sensitive hydrogel to deliver unmethylated Toll-like receptor agonist-9 (TLR9) in the bilateral inguinal area. Cytosine-phosphate-guanine oligonucleotide (CpG-ODN) is used as an immune adjuvant to induce anti-tumor immunity. Tumor cells are well suppressed in the environment of direct killing chemotherapy combined with immune response (Liu et al., 2024).

Multi strategy collaborative therapy seems to have more advantages than single strategy therapy. Multi directional treatment can effectively avoid the decrease in drug sensitivity of tumors and improve treatment effectiveness. In addition, this can also reduce excessive intake of a single drug and lower the toxic side effects of the drug on the body. Using hydrogel platform to achieve synergistic and powerful hydrogel preparations may be an effective solution for urothelial cancer.

## 4 Conclusion and prospects

With its excellent drug delivery ability, hydrogels have been extensively studied in situ drug delivery for the treatment of urothelial cancer, ranging from a single treatment strategy to a combination of multiple strategies. The advantage of the hydrogel drug delivery system that it can be processed and modified can correspondingly improve the current difficulties in situ drug delivery in the urinary system, thereby achieving functions that cannot be achieved by ordinary drug delivery systems. It is a promising treatment strategy. In view of the increasingly prominent problem of reduced tumor sensitivity to drugs, multi-strategy collaborative therapy seems to have greater advantages. However, currently there are very few hydrogel drug-loading systems that can be used clinically. Hydrogel drug-loading systems that combine high encapsulation efficiency, long sustained release time, strong penetration ability, and good targeting effect are the focus of current research and development. However, its safety and stability in clinical translation are issues that need to be solved urgently.
